# The Effects of Invisible Solar Rays

**Published:** 1921-05-14

**Authors:** 


### SCIENCE AND EYE STRAIN.

### The Effects of Invisible Solar Rays.

We have received the second post-war number of "Guy's Hospital Reports,"* and among many valuable contributions which the staff of this hospital has provided one particularly calls for the practical hygienist's attention. This is a paper by Mr. A. W. Ormond, C.B.E., F.R.C.S., ophthalmic surgeon to the hospital, on the pathological effects of the visible and invisible portions of the spectrum upon the human eye. Problems of illumination now occupy no little public attention, whether in connection with the glare of motor lamps, cinema films, the snow-blindness of the Arctic, or various industrial disabilities, so that it is with particular interest that we abstract some of an experienced ophthalmologist's findings.

### "Ether" Waves.

In the first place it is to be remembered that perceptible light rays form but a small central section of the true spectrum. Accepting the theory that the various forms of radiant energies are vibrations of the "ether," they appear to differ essentially in the length and rapidity of their vibrations. Thus, beginning with those of shortest wave length and progressing upwards, we have,

(1) *X*-rays.
(2) Ultra-violet and actinic (invisible).
(3) Violet, indigo, blue, green, yellow, orange and red lights.
(4) Infra-red; heat (invisible).
(5) The Hertzian waves (invisible).

Those of shortest wave length have the most rapid vibration speed; thus *x*-rays, at the top of the list, have a short wave length and high speed, and the waves used in wireless telegraphy (Hertzian) are the longest and, therefore, least rapid. One factor is common to them all, their rate of actual transmission through space is the same. As Mr. Ormond explains, however, the physiological and pathological effects of these rays are not so definite as their physical properties. There is some degree of overlapping, and to render explanation easier he therefore divides them simply into (*a*) ultra-violet (invisible), (*b*) light (visible), and (*c*) infra-red (invisible). Some important medical facts can then be stated. The conjunctiva, cornea, and lens absorb all the ultra-violet rays. The transparent media of the eye are uniformly permeable to so-called light rays, so that light passing through the pupil produces definite effects only on reaching the retinal pigment. Of the infra-red rays, which include the heat rays, a proportion only reach the retina, about 12 per cent. being stopped by the lens. Of those reaching the retina, the majority are absorbed by the retinal and choroidal pigment, but a small number actually reach the orbit, *i.e.*, pass through and beyond the globe of the eye.

* "Guy's Hospital Reports." Issued quarterly. Single numbers, 12s. 6d. Published by both Henry Frowde and Hodder and Stoughton, London.

Having outlined the fate of rays entering the eye, the next point is to examine the type of rays coming from sources encountered in life. The spectrum of a petroleum lamp contains fewer ultra-violet rays than that of an ordinary electric lamp and far less than the arc lamp or the sun. Ultra-violet rays are those having intense chemical activity, and they are also irritating to the eyes. Finsen and his assistants in their earlier experiments suffered from this cause. Their essential effect is either a stimulation of the nerve endings in the conjunctiva (the superficial membrane on the front of the eye) or a direct chemical irritation of the same area. But in any case, it is evident that any source of light having a high ultra-violet content is open to objection, and from this standpoint the modern electric varieties are not an improvement upon gas and oil.

### Ultra-Violet Rays.

Mr. Ormond finds, however, much more conclusive evidence of these ultra-violet effects in the phenomena of snow-blindness. Part of this observation is based upon a patient who formed part of a Canadian Arctic expedition, and he quotes this officer's own description of the symptoms. The eyes become bloodshot and slightly inflamed, while the lids feel as if grit had lodged in them. Within twelve hours this is felt all over the eyes, there is an urgent desire to close them and a clear discharge from both eyes and nose commences. Within twenty-four hours the pain is intense and headache becomes intolerable. Any voluntary effort to move the eyes is agonising, and the lids, once parted with the fingers, remain in any position in which they are put. Nothing can be seen distinctly even when the eyes are held open, but the sensation to light and darkness is still maintained. Gradual recovery takes place within seventy-two hours if care be taken, but thereafter the person has a decided tendency to relapse if unfavourable conditions are encountered. According to experience, the circumstances most likely to produce snow-blindness arise with the mist or fog in the early Arctic spring. Then, in the landscape, all sense of perspective is lost. On the other hand, when rocks and earth become uncovered and show as dark points snow-blindness is far less frequent, and one method of prevention is to carry before one in walking a dark object at the end of a stick.

As to which part of the spectrum is responsible for this form of optical harm, Mr. Ormond excludes the infra-red waves, as being heat waves and unlikely to be troublesome in the Arctic. The visible spectrum is clearly not responsible, because in the tropics, where this light is intense, the same kind of blindness is not met with. Thus we are left with the ultra-violet rays as the most likely cause.

In support of the idea it is noteworthy that symptoms of snow-blindness can be artificially produced. During the war a method of signalling at sea was invented, whereby all except the ultra-violet rays of a searchlight were cut off. The message was then received on a fluorescent screen, which became luminous when the invisible rays fell upon it. If one works for any length of time with these rays a condition analogous to snow-blindness is produced. There are other examples, but in any case it seems fairly certain that the ultra-violet rays or invisible rays at the blue end of the spectrum are definitely irritating. According to Scott and Shackleton animals are equally susceptible, but the query naturally arises as to why snow-blindness does not occur in, say, Russia. Apparently it is a question of quantity as well as quality of the rays. Ultra-violet rays are markedly absorbed by denser atmospheres.

The effects of entry of excess of luminous rays to the eye are well enough known in this country. Mr. Ormond records a case of temporary damage to vision through attempts to observe a certain solar phenomenon through smoked glass. In practice varying degrees of damage occur according to the brilliance of the light and the time of exposure. Many possible standards of luminosity might be quoted, but apparently the permissible brilliancy for comfort—*e.g.*, reading—is about two to three candle-power to a square inch. It is in industrial life, however, that the less known optical effects are produced and this by the infra-red or heat rays. Glass-workers, handling molten highly luminous material, are proportionately very liable to cataract. As already explained, a number of these rays are stopped or absorbed by the lens of the eye. In this work the intense light causes the pupil to contract, and the heat rays act, therefore, upon the central part only of the lens. The first sign of cataract appears, therefore, in this area, and although it takes many years for the effect to be produced, ultimately the area of opacity slowly extends. There has been some uncertainty as to which ray really causes "glass-worker's cataract," but Mr. Ormond puts forward a strong argument in favour of the infra-red. Whichever ray is responsible, the immediate cause is, of course, coagulation by the rays of the protein in the lens itself.

### Protective Measures.

From these notes it is now obvious how certain apparently perfectly transparent glasses exert their protective effect upon the eyes. No effort has been more successful than that of Sir William Crookes in devising formulæ for different varieties of glass to meet various conditions. One kind, to protect glass-blowers, succeeds in cutting off 96 per cent. of the heat and transmits 40 per cent. of the light rays. Another Crookes glass can eliminate the ultra-violet, cut off 37 per cent. of the heat and yet transmit 99.5 per cent. of the light. There are many more, but the above will serve to illustrate the optical principle, and in conclusion, Mr. Ormond draws attention to the fact that it is not only the rays forming the visible spectrum (light as we know it), but also those forming the invisible spectrum, which can and do produce certain pathological changes and "strains" in our eyes. It is a fascinating subject, and further elucidation of the exact mechanism by which such optical damage is done should well repay the time and trouble spent.

